# Multifunctional Phytocompounds in *Cotoneaster* Fruits: Phytochemical Profiling, Cellular Safety, Anti-Inflammatory and Antioxidant Effects in Chemical and Human Plasma Models *In Vitro*

**DOI:** 10.1155/2018/3482521

**Published:** 2018-10-24

**Authors:** Agnieszka Kicel, Joanna Kolodziejczyk-Czepas, Aleksandra Owczarek, Magdalena Rutkowska, Anna Wajs-Bonikowska, Sebastian Granica, Pawel Nowak, Monika A. Olszewska

**Affiliations:** ^1^Department of Pharmacognosy, Faculty of Pharmacy, Medical University of Lodz, 1 Muszynskiego, 90-151 Lodz, Poland; ^2^Department of General Biochemistry, Faculty of Biology and Environmental Protection, University of Lodz, Pomorska 141/143, 90-236 Lodz, Poland; ^3^Institute of General Food Chemistry, Faculty of Biotechnology and Food Sciences, Lodz University of Technology, 4/10 Stefanowskiego, 90-924 Lodz, Poland; ^4^Department of Pharmacognosy and Molecular Basis of Phytotherapy, Faculty of Pharmacy, Medical University of Warsaw, 1 Banacha, 02-097 Warsaw, Poland

## Abstract

The work presents the results of an investigation into the molecular background of the activity of *Cotoneaster* fruits, providing a detailed description of their phytochemical composition and some of the mechanisms of their anti-inflammatory and antioxidant effects. GS-FID-MS and UHPLC-PDA-ESI-MS^3^ methods were applied to identify the potentially health-beneficial constituents of lipophilic and hydrophilic fractions, leading to the identification of fourteen unsaturated fatty acids (with dominant linoleic acid, 375.4–1690.2 mg/100 g dw), three phytosterols (with dominant *β*-sitosterol, 132.2–463.3 mg/100 g), two triterpenoid acids (10.9–54.5 mg/100 g), and twenty-six polyphenols (26.0–43.5 mg GAE/g dw). The most promising polyphenolic fractions exhibited dose-dependent anti-inflammatory activity in *in vitro* tests of lipoxygenase (IC_50_ in the range of 7.7–24.9 *μ*g/U) and hyaluronidase (IC_50_ in the range of 16.4–29.3 *μ*g/U) inhibition. They were also demonstrated to be a source of effective antioxidants, both in *in vitro* chemical tests (DPPH, FRAP, and TBARS) and in a biological model, in which at *in vivo*-relevant levels (1–5 *μ*g/mL) they normalized/enhanced the nonenzymatic antioxidant capacity of human plasma and efficiently protected protein and lipid components of plasma against peroxynitrite-induced oxidative/nitrative damage. Moreover, the investigated extracts did not exhibit cytotoxicity towards human PMBCs. Among the nine *Cotoneaster* species tested, *C. hjelmqvistii*, *C. zabelii*, *C. splendens*, and *C. bullatus* possess the highest bioactive potential and might be recommended as dietary and functional food products.

## 1. Introduction

Edible fruits are widely recognized as a valuable source of structurally diverse phytochemicals with a broad spectrum of health-promoting properties. Decreased cholesterol levels, lower blood pressure, better mental health, and protection against cancer are only a few of the many benefits associated with the regular intake of fruit products, as indicated by numerous epidemiological studies [1]. Among the different fruit-bearing families, the Rosaceae seems to be of special importance. With over 3000 species, the family provides numerous types and varieties of fruits, some of which, such as apples, pears, strawberries and cherries, have great economic and dietary importance, and are frequently and willingly consumed due to their excellent flavors and proven nutritional value [2]. Many other taxa (e.g., *Aronia* sp., *Sorbus* sp., *Pyracantha* sp., and *Prunus spinosa* L.) produce fruits, that while less attractive in taste and appearance, are, nonetheless, distinguished by especially high quantities of bioactive constituents, which makes them perfect candidates for more specialized food applications, for example, as functional food products or food additives [3–6].

The chemical diversity of health-beneficial phytochemicals contained in rosaceous plant materials is immense and ranges from highly lipophilic to strongly polar constituents. Unsaturated fatty acids of almond oil, the cholesterol-regulating phytosterols of *Prunus africana* (Hook.f.) Kalkman, and the pentacyclic triterpenes, ubiquitous throughout the Rosaceae, with proven anti-inflammatory activity are some examples of the possible structures from the hydrophobic end of the spectrum [7, 8]. On the other hand, the hydrophilic fractions often contain an abundance of highly-valued polyphenol antioxidants belonging to numerous chemical classes, such as flavonoids, phenolic acids, and tannins. The bioactive potential of Rosaceae fruits is, therefore, associated not with a single fraction but rather is an effect of the presence of a range of phytochemicals.

**Figure 1:**
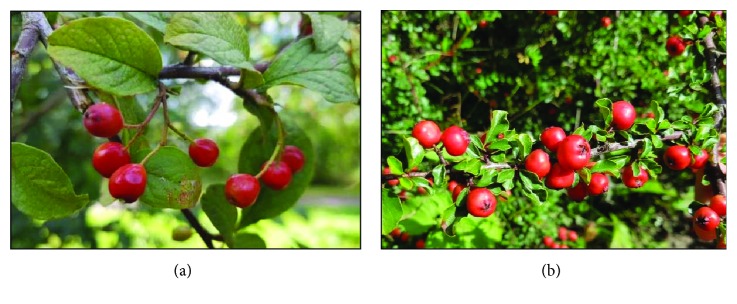
The fruits of *C. bullatus* (a) and *C. splendens* (b).

The genus *Cotoneaster* Medikus is one of the largest genera of the Rosaceae family (subfamily Spiraeoideae, tribe Pyreae) comprising about 500 species of shrubs or small trees. Its members are native to the Palearctic region (temperate Asia, Europe, north Africa) but are often cultivated throughout Europe as ornamental plants due to their decorative bright red fruits (Figure 1). The center of diversity of the taxon are the mountains of southwestern China and the Himalayas [9, 10], where the fruits have been used for culinary purposes by the local communities. The nutritional value of the fruits as a source of vitamins and minerals has been confirmed [11, 12] and additional beneficial health effects of the fruit consumption have been also reported in the traditional medicine for the treatment of diabetes mellitus, cardiovascular diseases, nasal hemorrhage, excessive menstruation, fever, and cough [9, 10]. The phytochemical research on the subject is scarce, but the available data indicate the tendency of the fruits to accumulate a wide range of active metabolites. In particular, the fruits of *Cotoneaster pannosus* Franch. are a source of linoleic acid, those of *Cotoneaster microphylla* Wall ex Lindl contain pentacyclic triterpenoids, and the polyphenolic fractions of *C. pannosus* and *Cotoneaster integerrimus* Medik. fruits are rich in epicatechin, shikimic acid, and chlorogenic acid [9, 11, 12]. However, broader generalization of their properties is troublesome, and the possible wider application of the fruits, for example, as functional food products, is hindered by a lack of systematic studies. Similarly limited is the information on the activity of *Cotoneaster* fruits. Preliminary studies have been performed on the fruits of *C. integerrimus* and *C. pannosus* with regard to their antioxidant, anticholinesterase, antityrosinase, antiamylase, and antiglucosidase properties, and their free radical-scavenging potential was proven to be the most promising [9, 12]. Still, the research was carried out using only simple *in vitro* chemical tests and did not cover *in vivo*-relevant antioxidant mechanisms.

The aim of this study was, therefore, to provide a more detailed insight into the chemical composition and activity of *Cotoneaster* fruits. To this end, the fruits from nine species of *Cotoneaster* cultivated in Poland were analyzed for a range of lipophilic and hydrophilic (polyphenolic) constituents with acknowledged health-promoting properties using a combination of chromatographic and spectroscopic methods (GC-FID-MS, UHPLC-PDA-ESI-MS^3^, and UV-Vis spectrophotometry). The most promising polyphenolic fractions were then subjected to an analysis of antioxidant activity comprising eight complementary *in vitro* tests (both chemical and biological plasma models) covering some of the mechanisms crucial for reducing the level of oxidative damage in the human organism, that is, scavenging of free radicals, enhancement of the nonenzymatic antioxidant capacity of blood plasma, and protection of its lipid and protein components against oxidative/nitrative changes. Additionally, the inhibitory effects of the fruit extracts on the proinflammatory enzymes, that is, lipoxygenase and hyaluronidase, were also measured. Finally, the cellular safety of the extracts was evaluated in cytotoxicity tests employing human peripheral blood mononuclear cells (PMBCs).

## 2. Materials and Methods

### 2.1. Plant Material

The fruit samples of nine selected *Cotoneaster* Medik. species, that is, *C. lucidus* Schltdl. (AR), *C. divaricatus* Rehder et E.H. Wilson (BG), *C. horizontalis* Decne. (BG), *C. nanshan* Mottet (BG), *C. hjelmqvistii* Flinck et B. Hylmö (BG), *C. dielsianus* E. Pritz. (BG), *C. splendens* Flinck et B. Hylmö (BG), *C. bullatus* Bois (BG), and *C. zabelii* C.K. Schneid. (BG) were collected in September 2013, in the Botanical Garden (BG; 51°45′N 19°24′E) in Lodz (Poland) and in the Arboretum (AR; 51°49′N 19°53′E), Forestry Experimental Station of Warsaw University of Life Sciences (SGGW) in Rogow (Poland). The voucher specimens were deposited in the Herbarium of the Department of Pharmacognosy, Medical University of Lodz (Poland). The raw materials were powdered with an electric grinder, sieved through a 0.315 mm sieve, and stored in airtight containers until use.

### 2.2. General

Reagents and standards of analytical or HPLC grade such as 2,2-diphenyl-1-picrylhydrazyl (DPPH), 2,4,6-tris-(2-pyridyl)-s-triazine (TPTZ), 2,2′-azobis-(2-amidinopropane)-dihydrochloride (AAPH), linoleic acid, 2-thiobarbituric acid, Tween® 40, 5,5′-dithiobis-(2-nitrobenzoic acid) (DNTB), xylenol orange disodium salt, Histopaque®-1077 medium *N*,*O*-*bis*-(trimethylsilyl)-trifluoroacetamide with 1% 1-trimethylchlorosilane (BSTFA + TMCS), boron trifluoride, bovine testis hyaluronidase, lipoxygenase from soybean, reference standards of fatty acid methyl esters (FAMEs), ethyl oleate, 5-*α*-cholesterol, (±)-6-hydroxy-2,5,7,8-tetramethylchromane-2-carboxylic acid (Trolox®), butylated hydroxyanisole (BHA), 2,6-di-*tert*-butyl-4-methylphenol (BHT), gallic acid monohydrate, quercetin dehydrate, chlorogenic acid hemihydrate (5-*O*-caffeoylquinic acid), 3-*O*- and 4-*O*-caffeoylquinic acids, hyperoside semihydrate, isoquercitrin, rutin trihydrate, procyanidins B-2 and C-1, (−)-epicatechin, and indomethacin were purchased from Sigma-Aldrich (St. Louis, MO, USA). The standards of quercetin 3-*O*-*β*-D-(2^″^-*O*-*β*-D-xylosyl)-galactoside and quercitrin (quercetin 3-*O*-*α*-L-rhamnoside) have previously been isolated in our laboratory from *C. bullatus* and *C. zabelii* leaves with at least 95% HPLC purity (unpublished results). A (Ca^2+^ and Mg^2+^)-free phosphate buffered saline (PBS) was purchased from Biomed (Lublin, Poland). Peroxynitrite was synthesized according to Pryor et al. [13]. Anti-3-nitrotyrosine polyclonal antibody, biotin-conjugated secondary antibody, and streptavidin/HRP were purchased from Abcam (Cambridge, UK). HPLC grade solvents such as acetonitrile and formic acid were from Avantor Performance Materials (Gliwice, Poland). For chemical tests, the samples were incubated at a constant temperature using a BD 23 incubator (BINDER, Tuttlingen, Germany) and measured using a UV-1601 Rayleigh spectrophotometer (Beijing, China). Activity tests in blood plasma models and enzyme inhibitory assays were performed using 96-well plates and monitored using a SPECTROStar Nano microplate reader (BMG LABTECH, Ortenberg, Germany).

### 2.3. Phytochemical Profiling

#### 2.3.1. Extraction and Derivatization of Lipophilic Phytochemicals

The fruit samples (7.0 g) were exhaustively extracted in a Soxhlet apparatus with chloroform (150 mL, 24 h), to give lipid extracts (288–467 mg dw), which were then subjected to quantification of lipophilic compounds. Fatty acids were assayed as fatty acid methyl esters (FAMEs) prepared according to a method described earlier [14]. Phytosterols and triterpenes were assayed after their transformation to trimethylsilyl ethers (TMSs) according to Thanh et al. [15]. The FAME and TMS mixtures were independently analyzed by GC-FID-MS.

#### 2.3.2. GC-FID-MS Analysis

The analyses of lipophilic fractions were performed on a Trace GC Ultra instrument coupled with a DSQII mass spectrometer (Thermo Electron, Waltham, MA, USA) and a MS-FID splitter (SGE Analytical Science, Trajan Scientific Americas, Austin, TX, USA). The applied mass range was 33–550 amu, ion source-heating was 200°C, and ionization energy was 70 eV. The conditions for FAMEs were as follows: capillary column: TG-WaxMS (30 m × 0.25 mm i.d., film thickness 0.25 *μ*m; Thermo Fisher Scientific, Waltham, MA, USA); temperature program: 3–30 min: 50–240°C at 4°C/min; and injector and detector temperatures: 250°C and 260°C, respectively. The conditions for TMSs were as follows: capillary column: HP-5 (30 m × 0.25 mm i.d., film thickness 0.25 *μ*m; Agilent Technologies, Santa Clara, CA, USA); temperature program: 1–15 min: 100–250°C, at 10°C/min; 15–30 min: 250–300°C, at 4°C/min; and injector and detector temperatures: 310°C and 300°C, respectively. In all cases, the carrier gas was helium (constant pressure: 300 kPa). The lipophilic analytes were identified by comparison of their MS profiles with those stored in the libraries NIST 2012 and Wiley Registry of Mass Spectral Data (10th and 11th eds). Retention times (*t*_*R*_) of FAMEs were also compared with those of the commercial FAME mixture. The analyte levels were expressed as mg/100 g fruit dry weight (dw), calculated using the internal standards of ethyl oleate and 5-*α*-cholesterol (for the fatty acids as well as phytosterols and triterpenoids, respectively) and it was recalculated to the content in the plant material taking into account the extraction yield.

#### 2.3.3. Extraction of Polyphenolic Compounds

The fruit samples (100–500 mg) were first defatted by preextraction with chloroform (20 mL, 15 min; the chloroform extracts were discarded), then refluxed for 30 min with 30 mL of 70% (*v*/*v*) aqueous methanol, and twice for 15 min with 20 mL of the same solvent. The combined extracts were diluted with the extractant to 100 mL. Each sample was extracted in triplicate to give the test extracts, which were analyzed for their total phenolic contents (TPCs) and antioxidant activity in chemical models. For UHPLC analyses and antioxidant activity evaluation in the human plasma models, the test extracts were evaporated *in vacuo* and lyophilized using an Alpha 1-2/LDplus freeze dryer (Christ, Osterode am Harz, Germany) before weighing.

#### 2.3.4. UHPLC-PDA-ESI-MS^3^ Analysis

Metabolite profiling was performed on an UltiMate 3000 RS UHPLC system (Dionex, Dreieich, Germany) with PDA detector scanning in the wavelength range of 220–450 nm and an amaZon SL ion trap mass spectrometer with ESI interface (Bruker Daltonics, Bremen, Germany). Separations were carried out on a Kinetex XB-C18 column (150 × 2.1 mm, 1.7 *μ*m; Phenomenex Inc., Torrance, CA, USA). The mobile phase consisted of solvent A (water-formic acid, 100 : 0.1, *v*/*v*) and solvent B (acetonitrile-formic acid, 100 : 0.1, *v*/*v*) with the following elution profile: 0–45 min, 6–26% (*v*/*v*) B; 45–55 min, 26–95% B; 55–60 min, 95% B; and 60–63 min, 95–6% B. The flow rate was 0.3 mL/min. The column temperature was 25°C. Before injections, samples of dry extracts (15 mg) were dissolved in 1.5 mL of 70% aqueous methanol, filtered through PTFE syringe filters (25 mm, 0.2 *μ*m, Vitrum, Czech Republic) and injected (3 *μ*L) into the UHPLC system. UV-Vis spectra were recorded over a range of 200–600 nm, and chromatograms were acquired at 280, 325, and 350 nm. The LC eluate was introduced directly into the ESI interface without splitting and analyzed in a negative ion mode using a scan from *m*/*z* 70 to 2200. The MS^2^ and MS^3^ fragmentations were obtained in Auto MS/MS mode for the most abundant ions at the time. The nebulizer pressure was 40 psi, dry gas flow was 9 L/min, dry temperature was 300°C, and capillary voltage was 4.5 kV.

#### 2.3.5. Determination of Total Phenolic Content (TPC)

The TPC levels were determined according to the Folin-Ciocalteu method as described previously [16]. The results were expressed as mg of gallic acid equivalents (GAE) per g of dry weight of the plant material (mg GAE/g dw).

### 2.4. Lipoxygenase (LOX) and Hyaluronidase (HYAL) Inhibition Tests

The ability of the fruit extracts to inhibit lipoxygenase (LOX) and hyaluronidase (HYAL) was evaluated according to the method optimized earlier [17]. The results of both tests were expressed as IC_50_ values (*μ*g/mL) from concentration-inhibition curves.

### 2.5. Antioxidant Activity in Chemical Models

The DPPH free-radical scavenging activity was determined according to a previously optimized method [16] and expressed as normalized EC_50_ values calculated from concentration-inhibition curves. The FRAP (ferric reducing antioxidant power) was determined according to [16] and expressed in *μ*mol of ferrous ions (Fe^2+^) produced by 1 g of the dry extract or standard, which was calculated from the calibration curve of ferrous sulfate. The ability of the extracts to inhibit AAPH-induced peroxidation of linoleic acid was assayed as described previously [18] with peroxidation monitored by quantification of thiobarbituric acid-reactive substances (TBARS) according to a previously optimized method [19], and the antioxidant activity was expressed as IC_50_ values calculated from concentration-inhibition curves. Additionally, the activity parameters in all of the assays were also expressed as *μ*mol Trolox® equivalents (TE) per g of dry weight of the plant material (*μ*mol TE/g dw).

### 2.6. Antioxidant Activity in Human Plasma Models

#### 2.6.1. Isolation of Blood Plasma and Sample Preparation

Blood (buffy coat units) from eight healthy volunteers, received from the Regional Centre of Blood Donation and Blood Treatment in Lodz (Poland), was centrifuged to obtain plasma [20]. All experiments were approved by the committee on the Ethics of Research at the Medical University of Lodz RNN/347/17/KE. Plasma samples, diluted with 0.01 M Tris/HCl pH 7.4 (1 : 4 *v*/*v*), were preincubated for 15 min at 37°C with the examined extracts, added to the final concentration range of 1–50 *μ*g/mL, and then exposed to 100 or 150 *μ*M peroxynitrite (ONOO^−^). Control samples were prepared with plasma untreated with the extracts and/or peroxynitrite. To eliminate the possibility of direct interactions of the extracts with plasma proteins and lipids, several experiments with blood plasma and the extracts only (without adding ONOO^−^) were also performed and no prooxidative effect was found.

#### 2.6.2. Determination of 3-Nitrotyrosine and Thiols in Human Plasma Proteins

The peroxynitrite-induced protein damage in blood plasma was determined by the use of 3-nitrotyrosine and protein thiol levels (−SH) as biomarkers of oxidative stress. Immunodetection of 3-nitrotyrosine-containing proteins by the competitive ELISA (C-ELISA) method in plasma samples (control or antioxidants and 100 *μ*M ONOO^−^-treated plasma) was performed according to [20]. The nitrofibrinogen (3NT-Fg, at a concentration of 0.5 *μ*g/mL and 3–6 mol nitrotyrosine/mol protein) was prepared for use in the standard curve. The concentrations of nitrated proteins that inhibit antinitrotyrosine antibody binding were estimated from the standard curve and are expressed as the 3NT-Fg equivalents (in nmol/mg of plasma protein). The concentration of free thiol groups (−SH) in plasma samples (control or antioxidants and 100 *μ*M ONOO^−^-treated plasma) was measured spectrophotometrically according to Ellman's method [20]. The free thiol group concentration was calculated from the standard curve of glutathione (GSH) and expressed as umol/mL of plasma.

#### 2.6.3. Determination of Lipid Hydroperoxides and TBARS in Human Blood Plasma

The peroxynitrite-induced lipid peroxidation in blood plasma was determined spectrophotometrically by evaluation of the level of lipid hydroperoxides and TBARS. The concentration of hydroperoxides in plasma samples (control or antioxidants and 100 *μ*M ONOO^−^-treated plasma) was determined by a ferric-xylenol orange (FOX-1) protocol with a later modification [20]. The amount of lipid hydroperoxides was calculated from the standard curve of hydrogen peroxide and expressed in nmol/mg of plasma proteins. Determination of TBARS in plasma samples (control or antioxidants and 100 *μ*M ONOO^−^-treated plasma) was performed according to [20]. The TBARS values were expressed in *μ*mol TBARS/mL of plasma.

#### 2.6.4. Ferric Reducing Ability of Human Blood Plasma (FRAP)

The influence of the extracts on the nonenzymatic antioxidant status of plasma was conducted by measurements of their ability to reduce ferric ions (Fe^3+^) to ferrous ions (Fe^2+^). The experiments were performed according to Benzie and Strain [21] and modified by Kolodziejczyk-Czepas et al. [20]. The FRAP values of plasma samples (control or antioxidants and 150 *μ*M ONOO^−^-treated plasma) were expressed in mM Fe^2+^ in plasma as calculated from the calibration curve of ferrous sulphate.

### 2.7. Cellular Safety Testing

The cytotoxicity of the examined extracts was conducted in an experimental system of peripheral blood mononuclear cells (PBMCs). PBMCs were isolated from fresh human blood using the Histopaque®-1077 medium, according to a procedure described in our previous work [19]. Then, the cells (1 × 10^6^ PBMCs/mL, suspended in PBS) were incubated with *Cotoneaster* fruit extracts at the final concentrations of 5, 25, and 50 *μ*g/mL. Measurements of cell viability were executed after two, four, and six hours of incubation (at 37°C) in a routine dye excluding test, based on a staining with 0.4% Trypan blue. The procedure was carried out according to the manufacturer's protocol using a microchip-type automatic cell counter Bio-Rad (Hercules, CA, USA).

### 2.8. Statistical and Data Analysis

The statistical analysis was performed using STATISTICA 13Pl software for Windows (StatSoft Inc., Krakow, Poland). The results were reported as means ± standard deviation (SD) or ±standard error (SE) for the indicated number of experiments. The significance of differences between the samples and controls were analyzed by one-way ANOVA, followed by the post hoc Tukey's test for multiple comparison. A level of *p* < 0.05 was accepted as statistically significant.

## 3. Results and Discussion

### 3.1. GC-FID-MS Analysis of Fatty Acids

**Figure 2:**
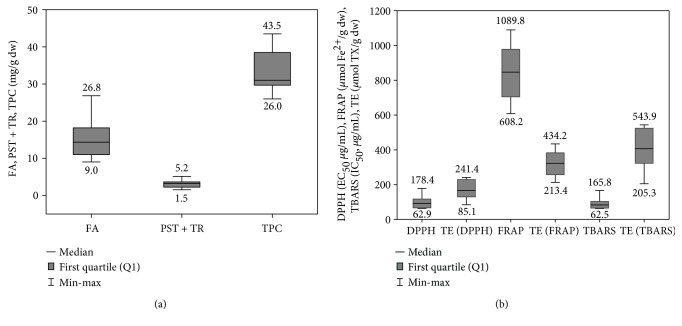
Variability of the measured quantitative and activity parameters among the investigated *Cotoneaster* fruits. (a) FA, total fatty acids; PS + TR, sum of phytosterols and tritrepenes; TPC, total phenolic content, expressed in gallic acid equivalents (GAE). (b) DPPH, radical scavenging activity expressed as EC_50_ value; FRAP, ferric reducing antioxidant power; TBARS, inhibition of linoleic acid peroxidation; TE, Trolox® equivalent antioxidant activity.

The fatty acid profiles of the lipophilic fractions in the chloroform extracts of the *Cotoneaster* fruits were determined by GC-FID-MS analysis of methyl ester derivatives (FAMEs). As shown in Table 1 and Figure 2, fourteen fatty acids were identified, including saturated, mono-, and polyunsaturated acids with chain lengths ranging from 6 to 22 carbon atoms. Their total content (TFA) varied among the *Cotoneaster* species from 902.5 to 2683.8 mg/100 g of fruit dry weight (dw) with the highest levels noted for *C. zabelii* (2683.8 mg/100 g dw) and *C. splendens* (2024.1 mg/100 g dw). All analyzed fruits contained primarily poly- and monounsaturated acids, constituting 41.6–66.8% and 18.6–29.6% of TFA, respectively. The major component in each sample was linoleic acid C18 : 2 Δ^9,12^, the sole representative of the polyunsaturated acids. Its content varied among species from 375.4 to 1690.2 mg/100 g fruit dw with the highest amounts (above 10 mg/g dw) recorded for the fruits of *C. zabelii*, *C. splendens*, *C. hjelmqvistii*, and *C. horizontalis*. Relatively high levels of oleic acid C18 : 1 Δ^9^, a monounsaturated acid, were also noted, especially for the *C. zabelii* and *C. splendens* (649.7 and 473.7 mg/100 g dw, respectively). Regarding saturated acids, they accounted for only 12.3–28.8% of TFA. The highest content of this group was observed in the fruits of *C. zabelii*, *C. splendens*, and *C. nanshan*, with palmitic acid C16 : 0 being the dominant compound (226.5, 212.6 and 168.7 mg/100 g dw, respectively).

The present work is the first comparison of several *Cotoneaster* fruits in terms of their fatty acid profile. Despite some quantitative differences observed between the investigated fruits, a high level of consistency can be noticed in the qualitative composition of this fraction. The results are in accordance with previous reports for the fruits of *C. pannosus* from Italy, as well as the branches of *C. horizontalis* Decke. of Egyptian origin and the seeds of *C. bullatus*, *C. dielsianus*, *C. francheti* Bois, *C. moupinensis* Franch., and *C. simonsii* Baker cultivated in Germany, in which linoleic and palmitic acids were also detected as the major fatty acid components [9, 22, 23].

The unsaturated fatty acids are known factors associated with the prevention of various chronic and acute diseases, such as cardiovascular diseases, osteoporosis, immune disorders, and cancer [7]. Linoleic acid, the representative of the omega-6 fatty acid family (essential fatty acids (EFA)), is considered a vital constituent of a healthy human diet, due to its contribution to cholesterol metabolism (regulation of plasma total cholesterol and low-density lipoprotein cholesterol levels and HDL-LDL ratio) and its association with a lower risk of atherosclerosis [24]. Main sources of this compound are plant oils, derived, inter alia, from the seeds of safflower, sunflower, grape, pumpkin, and corn. The available literature data [25, 26] indicate that whole fruits of some Rosaceae members, such as *Crataegus monogyna* Jacq., *Prunus spinosa* L., and *Rubus ulmifolius* Schott., might be considered as abundant in linoleic acid, constituting over 10% of their lipophilic fraction [26]. Our present results indicate that the analyzed *Cotoneaster* fruits also deserve more attention as rich sources of this compound.

### 3.2. GC-FID-MS Analysis of Phytosterols and Triterpenoids

Apart from fatty acids, three phytosterols (campesterol, *β*-sitosterol, and stigmasterol) and four triterpenes (*α*- and *β*-amyrins, ursolic and oleanolic acids) were identified in the chloroform extracts of the *Cotoneaster* fruits, based on GC-FID-MS analysis of their trimethylsilyl ether derivatives (TMSs). As reported in Table 2 and Figure 2, the total content of sterols and triterpenoids, depending on the tested species, was in the range of 154.6–515.6 mg/100 g of fruit (dw) with the highest levels observed for *C. splendens* (515.6 mg/100 g dw) and *C. nanshan* (438.0 mg/100 g dw). The dominant compound in all samples was *β*-sitosterol, with the levels ranging from 132.2 to 463.3 mg/100 g dw (76.5–89.3% of the total sterols and triterpenes). The highest content of *β*-sitosterol was observed for the fruit of *C. splendens* (463.3 mg/100 g dw) followed by those of *C. nanshan* (391.3 mg/100 g dw) and *C. horizontalis* (316.3 mg/100 g dw). Other individual components were observed at much lower concentrations, reaching at most 42 mg/100 g dw.

Regarding the phytosterol and triterpenoid profile, the present results are generally similar to the data obtained previously for different organs of *Cotoneaster* species, although some differences can be noticed in relative proportions of particular compounds. Among the sterols and triterpenoids identified earlier for the *C. horizontalis* branches collected in Egypt, *α*-amyrin was the dominant compound, constituting 14.4% of the total lipophilic constituents, followed by *β*-sitosterol (8.5%) and stigmasterol (1.1%) [23]. The ursolic acid was isolated previously from *C. simonsii* twigs [27], *C. racemiflora* Desf. twigs [28], and *C. microphylla* fruits [11], but the present work is the first to describe its quantitative levels in the *Cotoneaster* plants. On the other hand, betulinic acid, reported earlier for *C. microphylla* fruits [11], was not detected during the present study in any fruit sample.

Phytosterols (*β*-sitosterol, stigmasterol, and their analogues) are important dietary components which help regulate serum lipid profile, reduce total- and LDL-cholesterol levels, and increase HDL/LDL ratio. In addition, plant sterols possess anticancer, anti-inflammatory, and moderate antioxidant activities [29]. For instance, *β*-sitosterol, the most abundant plant sterol in the human diet, displays significant effects on reducing the symptoms of benign prostatic hyperplasia and prostate cancer. Moreover, this compound has been associated with antidiabetic, immunomodulatory, and analgesic properties [30]. Phytosterols are found abundantly in nonpolar fractions of plants, and their daily consumption is estimated in the range of 200–400 mg with the main dietary sources being vegetable oils, nuts, cereal products, vegetables, fruits, and berries [30]. They are also known to be present in abundance in the fruits derived from numerous genera of Rosaceae, including *Prunus*, *Crataegus*, and *Rosa* [25]. In the lipid fraction of rosaceous fruits, *β*-sitosterol was often identified as the predominant lipophilic compound, constituting usually more than 60% of the total sterols. As the daily intake of phytosterols (1.5–2.4 g) required for beneficial health effects, especially for cardiovascular and antiatherogenic protection, is usually higher than consumed with the common diet [30], dietary supplementation is a rational solution, and new plant sources of these biomolecules, such as the *Cotoneaster* fruits, offer promise in this aspect.

### 3.3. Polyphenolic Profiling of Fruit Extracts

**Figure 3:**
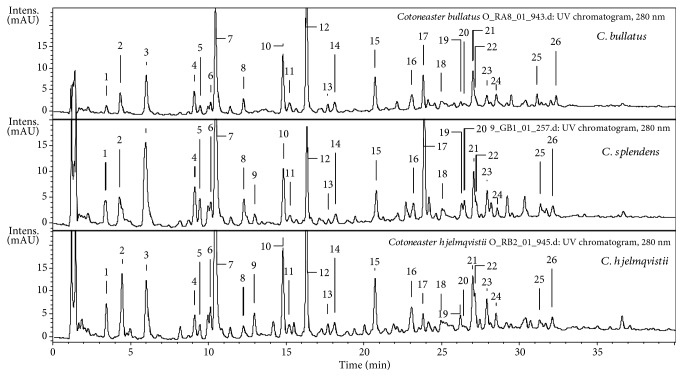
Representative UHPLC-UV chromatograms of the *C. bullatus*, *C. splendens*, and *C. hjelmqvistii* fruit polar extracts (*λ* = 280 nm). The peak numbers refer to those applied in Table 3.

LC-MS analysis of the hydrophilic (70% aqueous methanolic) extracts of the *Cotoneaster* fruits revealed the presence of a number of polyphenols (UHPLC peaks 1–26, Figure 3, Table 3) that were fully or tentatively identified by comparison of their chromatographic behavior and ESI-MS^3^ fragmentation pattern with authentic standards or literature values. Three major groups of polyphenols were recognized, including phenolic acids (3, 7, and 8) and their derivatives (1, 4, 5, and 11), flavan-3-ols including proanthocyanidins (9, 10, 12–16, 18, and 24), and flavonoids (17, 20, 21–23, 25, and 26). The recorded UHPLC fingerprints (Table 3) indicate that the phenolic profiles of all nine *Cotoneaster* fruits were qualitatively similar. However, noticeable differences were found in the proportions of individual polyphenols, which allowed the subgroups of species to be distinguished depending on the prevalent phenolic class. A distinctive feature of most *Cotoneaster* samples, especially *C. divaricatus*, *C. horizontalis*, and *C. nanshan*, was the predominance of phenolic acid derivatives (1, 3–5, 7, 8, and 11), mainly caffeoylquinic acids, with the dominant peak being chlorogenic acid (7). On the other hand, *C. zabelii*, *C. bullatus*, and *C. hjelmqvistii* contained relatively high amounts of flavan-3-ols and proanthocyanidins (9, 10, 12–16, 18, and 24), with dominating (−)-epicatechin (12). The contribution of flavonoids (17, 20, 21–23, 25, and 26) to the overall phenolic fraction was generally the lowest, but *C. splendens* was distinguished by a particularly large proportion of quercetin 3-(2^″^-xylosyl)-galactoside (17), and *C. dielsianus* contained a relatively higher level of hyperoside (21).

This report is the first comprehensive study of the LC-MS characteristics of the *Cotoneaster* fruits; the previous studies on *C. integerrimus* and *C. pannosus* have focused only on a selected aspect (HPLC-PDA) of their polyphenolic profiles [9, 12]. In contrast to the present results, the occurrence of low-molecular phenolic acids, including shikimic, *p*-coumaric, and benzoic acids, has been previously reported, and this phenomenon may be explained by the individual attributes of the tested samples or by differences in the methodology employed for the structural identification. On the other hand, the reported high level of (−)-epicatechin in the fruits of *C. integerrimus* [12] indicates its similarity to those of *C. zabelii* and *C. bullatus* analyzed in the present study.

The total phenolic content (TPC) of the 70% aqueous methanolic extracts of the *Cotoneaster* fruits was determined by the Folin-Ciocalteu photometric assay, commonly used to estimate phenolic metabolites as gallic acid equivalents (GAE). As shown in Table 4 and Figure 2, the TPC values in the analyzed fruits varied from 26.0 to 43.5 mg GAE/g of fruit dw. The highest phenolic content was found for the fruits of *C. hjelmqvistii* and *C. zabelii* (43.5 and 43.0 mg/g dw, respectively), followed by those of *C. splendens* and *C. bullatus* (38.5 and 37.3 mg/g dw, respectively). The level of phenolics in these species is comparable with those observed for other Rosaceae fruits reported in the literature as rich sources of natural polyphenols, for example, *Aronia melanocarpa* (Michx.) Elliott (34.4–78.5 mg GAE/g dw; [3]) and *Sorbus* species (22.4–29.8 mg GAE/g dw; [16]).

The presence of polyphenolic compounds in fruits and vegetables is strongly linked with the beneficial effects of these food products for human health, and the influence of polyphenols on closely intertwined processes of inflammation and oxidative stress is recognized as the most feasible mode of this action. As free radical scavengers, metal chelators, prooxidant and proinflammatory enzyme inhibitors, and modifiers of cell signaling pathways, polyphenols are effective agents preventing damages related to the oxidative stress and inflammation implicated in the etiology and progression of numerous chronic diseases, including cardiovascular diseases, diabetes mellitus, neurodegenerative disorders, and cancer [31–33]. The occurrence of polyphenolic compounds in the investigated fruits might thus largely define their bioactivity, especially that *Cotoneaster*-derived polyphenols have been previously linked with strong antioxidant capacity in our earlier study regarding the leaves [34].

### 3.4. Biological Activity

The above presented phytochemical studies proved that fruits of *Cotoneaster* species are indeed a rich source of diverse phytochemicals with a wide spectrum of recognized biological properties. However, based on the results of the quantitative studies, the polyphenolic fraction with the highest content would appear to have the greatest beneficial health effects of the fruits in a human organism. Thus, further studies were focused on providing a more detailed insight into potential mechanisms of the activity of the hydrophilic components, that is, their anti-inflammatory and antioxidant effects.

#### 3.4.1. Inhibitory Effects on Two Enzymes Involved in Inflammation

Inflammation is a complex process that constitutes a part of the immune system defense against harmful stimuli, but may lead to negative effects if uncontrolled. The inflammatory response is regulated by numerous enzymes and mediators and thus can be intercepted at different points, and several of these key enzymes, including lipoxygenases (LOX) and hyaluronidases (HYAL), are most often used to determine the anti-inflammatory potential of natural products [35]. LOX catalyze the diooxygenation of arachidonic acid to form hydroperoxides, the first step in the biosynthesis of several proinflammatory mediators [36]. HYAL, on the other hand, are highly specific hydrolases that degrade hyaluronic acid, an important component of the extracellular matrix, thus increasing the permeability of the tissues and facilitating the spread of inflammation [37]. Our present findings indicate that all fruit extracts inhibit the activity of LOX and HYAL in a dose-dependent manner (Table 5). The strongest inhibitory effect towards LOX was demonstrated by the leaf extracts of *C. hjelmqvistii* and *C. zabelii* (IC_50_ = 7.70 and 9.97 *μ*g/U, respectively), while the activity of HYAL was most strongly hindered by the leaf extract of *C. lucidus* (IC_50_ = 16.44 *μ*g/U). The activity of the extracts was weaker in comparison to indomethacin (IC_50_ = 1.89 *μ*g/U for LOX and 5.60 *μ*g/U for HYAL), but after recalculating the results to adjust for the actual polyphenol content (which gives IC_50_ values in the range of 0.33–0.77 *μ*g/U for LOX and 0.47–1.93 *μ*g/U for HYAL inhibition), the activity of the extracts looks quite advantageous in comparison to the positive standard. The anti-inflammatory potential of *Cotoneaster* polyphenols is further confirmed by the high activity of (−)-epicatechin, quercetin, and chlorogenic acid, the main constituents of the investigated leaf extracts.

#### 3.4.2. Antioxidant Activity in Chemical Models

The basic antioxidant mechanism of *Cotoneaster* polyphenols was verified in chemical models using three complementary *in vitro* assays: DPPH and FRAP tests, two of the most frequently employed SET (single electron transfer) type methods, and the inhibition of AAPH-induced linoleic acid peroxidation test (monitored by TBARS assay), a more physiologically relevant system which involves the HAT (hydrogen atom transfer) mechanism. In all of the applied tests, the investigated fruits displayed concentration-dependent activity with the capacity parameters (expressed in *μ*mol TE/g dw) of a similar order of magnitude, which shows that *Cotoneaster* antioxidants can effectively act via both basic mechanisms. The highest activity in comparison to the natural (quercetin) and synthetic standards (BHA and BHT) were observed in the FRAP and TBARS assays for all fruits (Table 4 and Figure 2). In all tests, the fruits of *C. zabelii*, *C. hjelmqvistii*, *C. bullatus*, and *C. splendens*, indicated in the present study as the richest sources of polyphenols, displayed the highest antioxidant efficiency, with the activity parameters varying in the narrow range of 225.5–240.9 *μ*mol TE/g dw (DPPH), 378.9–434.3 *μ*mol TE/g (FRAP), and 518.2–543.9 *μ*mol TE/g (TBARS), respectively. Interestingly, these were the species that also exhibited the relatively largest proportions of proanthocyanidins/flavan-3-ols (*C. zabelii*, *C. bullatus*, *C. splendens*) or quercetin 3-(2^″^-xylosyl)-glucoside (*C. hjelmqvistii*), which suggest that these polyphenols play a significant role in the activity of fruits. Additionally, the close connection between the phenolic levels and antioxidant parameters was also evidenced by statistically significant linear correlations between TPCs and the results of the DPPH (∣*r*∣ = 0.9352, *p* < 0.001), FRAP (∣*r*∣ = 0.9491, *p* < 0.001), and TBARS (∣*r*∣ = 0.9116, *p* < 0.001) tests.

#### 3.4.3. Protective Effects on Human Plasma Components Exposed to Oxidative Stress

To provide a more detailed insight into the antioxidant effects of *Cotoneaster* polyphenols, the four most promising species (*C. zabelii*, *C. bullatus*, *C. splendens*, and *C. hjelmqvistii*) were selected for further studies in a biological model. Since according to traditional application and our present results, *Cotoneaster* fruits appear to be promising sources of phytochemicals with properties especially advantageous for the circulatory system (i.e., linoleic acid and *β*-sitosterol), a human plasma model was selected to evaluate their additional benefits for cardiovascular health, this time mediated by polyphenols. This approach allowed for the *in vitro* monitoring of the protective effects of the extracts towards human plasma components under oxidative stress conditions. The peroxynitrite (ONOO^−^) used for inducing oxidative stress is a known *in vivo*-operating oxidant, responsible for structural changes in plasma proteins and lipids and implicated in numerous oxidative stress-related disorders [38]. The concentrations of ONOO^−^ (100 and 150 *μ*M) selected for the study enabled quantitative measurements of the resulting modifications in plasma components, but may be also regarded as physiologically-relevant as they can be reached *in vivo* in local compartments, for example, during a serious inflammation of blood vessels [39].

**Figure 4:**
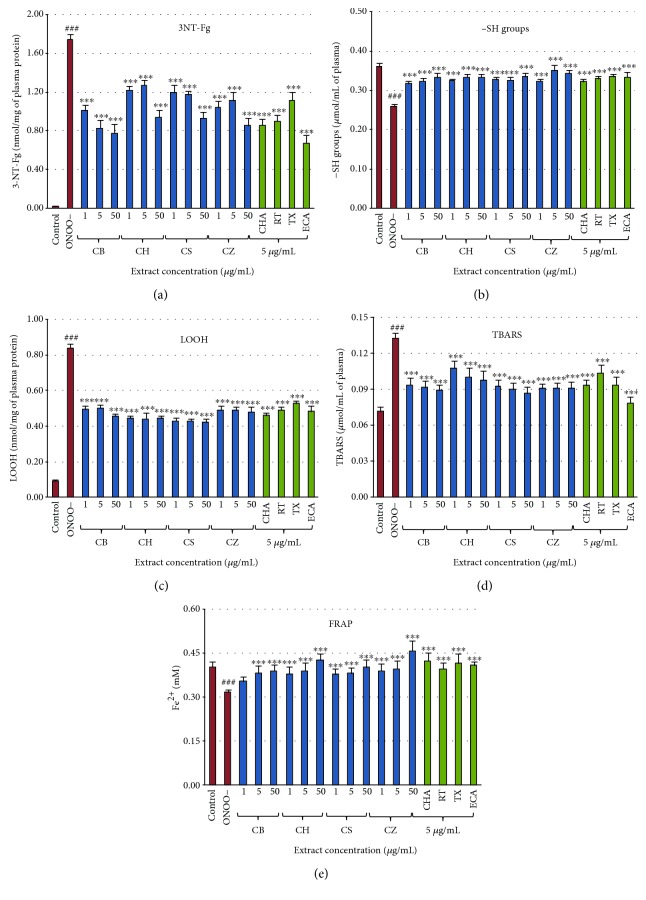
Effects of the *Cotoneaster* fruit extracts on human plasma exposed to oxidative stress: (a) effects on the nitration of tyrosine residues in plasma proteins and formation of 3-nitrotyrosine (3-NT-Fg); (b) effects on the oxidation of free thiol groups (−SH); effects on the peroxidation of plasma lipids including (c) formation of lipid hydroperoxides (LOOH), and (d) thiobarbituric acid-reactive substances (TBARS); (e) effects on ferric reducing ability of blood plasma (FRAP). Results expressed as means ± SE (*n* = 8) for repeated measures: ^###^*p* < 0.001, for ONOO^−^-treated plasma (without the extracts) versus control plasma, and ^∗∗∗^*p* < 0.001 for plasma treated with ONOO^−^ in the presence of the investigated extracts (1–50 *μ*g/mL) or the standards (5 *μ*g/mL). CB, *C. bullatus*; CH, *C. hjelmqvistii*; CS, *C. splendens*; CZ, *C. zabelii*. Standards: CHA, chlorogenic acid; RT, rutin; TX, Trolox®; ECA, (−)-epicatechin.

The addition of ONOO^−^ to the plasma samples resulted in an overall decrease (*p* < 0.001) in the nonenzymatic antioxidant capacity of the plasma, measured as the FRAP parameter, and in oxidative and nitrative alterations of its protein and lipid components, which was evidenced by a significant increase (*p* < 0.001) in lipid peroxidation biomarkers (lipid hydroperoxides and TBARS), a noticeable rise (*p* < 0.001) in 3-nitrotyrosine level (marker of protein nitration), and a decrease (*p* < 0.001) in the level of thiol groups (marker of protein oxidation). On the other hand, in the plasma samples incubated with ONOO^−^ in the presence of *Cotoneaster* extracts (1–50 *μ*g/mL), the extent of oxidative/nitrative damage to both proteins and lipids was noticeably limited (*p* < 0.05), regardless of the tested species and the extract concentration. As shown in Figures 4(a) and 4(b), even at the lowest concentrations of 1 *μ*g/mL, the extracts were able to reduce tyrosine nitration by about 29–42% and thiol group oxidation by about 24–26%, while at the concentration of 50 *μ*g/mL the effectiveness rose to 46–55% and 29–32%, respectively. Moreover, as demonstrated in Figures 4(c) and 4(d), all fruit samples inhibited the generation of plasma lipid hydroperoxides by 40–50% and reduced TBARS levels by 19–35%. All extract-treated samples, apart from those fortified with 1 *μ*g/mL of *C. bullatus* extract, demonstrated a statistically significant (*p* < 0.001) improvement in the nonenzymatic antioxidant capacity of blood plasma of up to 44% in comparison to the samples not protected by the extracts (Figure 4(e)). In most cases, little difference was observed in the activity between the tested fruits; however, the inhibition of tyrosine nitration assay found *C. bullatus* and *C. zabelii* displaying stronger activity than the other two extracts at all concentrations tested (*p* < 0.05). A dose dependency was noticeable for *C. bullatus* and *C. splendens* in antinitrative activity (Figure 4(a)) and for most *Cotoneaster* species in the TBARS test, with the exception of *C. zabelii* (Figure 4(d)). Some significant correlations were also found, between the TPCs and the activity parameters. The most prominent was the relationship for the FRAP assay (∣*r*∣ = 0.7587, *p* < 0.01). In the tests for protein protection, the correlation between the percentage inhibition of tyrosine nitration and phenolic level was stronger (∣*r*∣ = 0.6774, *p* < 0.05) than the analogous relationship for the reduction of thiol group oxidation (∣*r*∣ = 0.4885, *p* < 0.05). Contrastingly, the correlations in the lipid peroxidation assays were not statistically significant (*p* > 0.05).

The effectiveness of the extracts was further supported by the fact that in all of the tests, the observed antioxidant effects of the fruit extracts at the corresponding concentration levels (5 *μ*g/mL) were similar or higher to that of Trolox®, a synthetic analog of vitamin E often used as a positive standard in antioxidant studies. Moreover, the significant activity of rutin, chlorogenic acid, and, especially, (−)-epicatechin confirm the important role of polyphenols in the capacity of the extracts.

The wide range of the extract concentrations tested (1–50 *μ*g/mL) was in accordance with the general practice of *in vitro* studies [20] and allowed for the study of different interactions in the system. Additionally, the lower levels (1–5 *μ*g/mL) might be considered physiologically-relevant as they correspond to the levels of phenolics attainable *in vivo* after consumption of polyphenol-rich plant materials. For example, according to the accumulated research [40, 41], the maximal achievable concentration of plant phenolics in blood plasma can reach up to 5–10 *μ*M, which generally corresponds to less than 5 *μ*g/mL. Taking into account the TPC levels evaluated for *Cotoneaster* fruits in the present study and the extraction efficiency (15–30%, depending on the species), the levels of phenolics corresponding to the applied extract concentration of 1–5 *μ*g/mL are about 0.13–1.25 *μ*g/mL: well within the obtainable plasma range. This suggests that the protective activity of the *Cotoneaster* extracts towards ONOO^−^-induced changes observed *in vitro* may translate to their positive *in vivo* effects.

The harmful influence of ONOO^−^ is often associated with serious pathological consequences in many organs and systems of the human body. The nitration/oxidation of biomolecules such as enzymes, receptors, lipoproteins, fatty acids, or nucleic acids changes their function and may impair cellular signalization pathways, induce inflammatory responses, or even promote cell apoptosis [38, 39]. In the case of the circulatory system, the negative effects of ONOO^−^ result in a higher risk of cardiovascular disorders, such as stroke, myocardial infarction, or chronic heart failure [38], and are connected with the direct modifications of plasma proteins and lipids. For instance, the formation of 3-nitrotyrosine in fibrinogen might contribute to prothrombotic events in the blood coagulation cascade and fibrinolysis process [42], while thiol oxidation in platelet proteins leads to the inhibition of platelet function [43]. Additionally, oxidation of low-molecular-weight thiols, such as reduced glutathione, diminishes the endogenous antioxidant capacity of plasma and primes further oxidative damage in the system [38]. Similarly, lipid peroxidation initiated by ONOO^−^ may propagate platelet aggregation [44], while peroxynitrite-modified LDL binds with high affinity to macrophage scavenger receptors leading to foam cell formation, which represent a key early event in atherogenesis [38, 45]. The prevention of these processes partially explains the beneficial effects of *Cotoneaster* fruits reported by traditional medicine and might be regarded as a good strategy in prophylaxis of various cardiovascular complaints.

### 3.5. Cellular Safety

**Figure 5:**
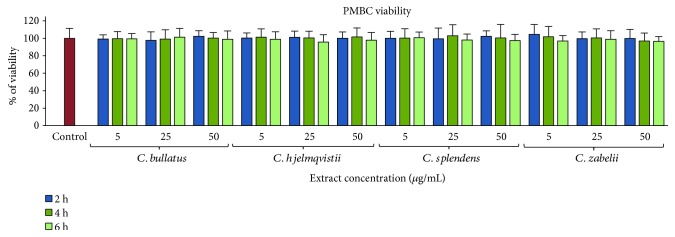
Viability of peripheral blood mononuclear cells (PMBCs) after 2, 4, and 6 h incubation with the *Cotoneaster* fruit extracts at 5, 25, and 50 *μ*g/mL. Results are presented as means ± SD (*n* = 14).

Due to its long tradition of consumption and application in folk medicine, the *Cotoneaster* fruits might be regarded as nontoxic. However, in the case of the concentrated extracts, a more detailed evaluation of their safety is required. Therefore, the next step of our research was a viability test on PMBCs which assessed the cytotoxicity of the extracts. After two, four, and six-hour incubation periods with the plant extracts at concentrations of 5, 25, and 50 *μ*g/mL, the viability of the extract-treated cells constituted 97.3–101.7% of that of the control (non-treated cells) and no statistically significant differences were found (*p* > 0.05) between the two values (Figure 5). These findings suggest that the *Cotoneaster* extracts do not have cytotoxic effects at these concentrations.

## 4. Conclusion

The current paper presents the first comprehensive phytochemical and activity study of *Cotoneaster* fruits. The fruits were found to possess distinct lipophilic and phenolic profiles, significant antioxidant activity in both chemical and biological models, noticeable inhibitory effects on the proinflammatory enzymes, and cellular safety. Hence, *Cotoneaster* fruits appear to be promising candidates for the production of pharma- and nutraceuticals associated with preventing and treating oxidative stress and inflammatory-related chronic diseases; they may also contribute to a balanced and varied diet comprising food rich in bioactive compounds. Furthermore, the protective effects against ONOO^−^-induced modifications in the plasma components, demonstrated by the polyphenolic fractions from the fruits of *C. hjelmqvistii*, *C. zabelii*, *C. splendens*, and *C. bullatus* at *in vivo*-relevant levels, may be considered as a molecular basis for the beneficial effects of *Cotoneaster* fruits within the cardiovascular system reported by traditional medicine. The biological activity demonstrated in the present study might therefore be a starting point of more extensive investigation on the nutritional value and bioactivity of *Cotoneaster* fruits, including their effects in *in vivo* systems.

## Figures and Tables

**Table 1:** Content of fatty acids (mg/100 g dw) in the *Cotoneaster* fruits.^a^

Fruit sample	6 : 0	8 : 0	12 : 0	14 : 0	15 : 0	16 : 0	17 : 0	16 : 1 Δ^9^	18 : 0	18 : 1 Δ^9^	20 : 0	18 : 2 Δ^9,12^	20 : 1 Δ^11^	22 : 0
*C. lucidus*	3.41 ± 0.10^B^	2.35 ± 0.10^F^	2.77 ± 0.01^F^	5.97 ± 0.30^E^	nd	126.25 ± 5.23^A^	tr	8.96 ± 0.51^E^	92.98 ± 5.12^F^	258.05 ± 12.11^A,B^	6.02 ± 0.20^A^	375.35 ± 18.01^A^	tr	18.77 ± 0.80^B^
*C. divaricatus*	2.24 ± 0.11^A^	0.61 ± 0.03^A,B^	0.82 ± 0.05^A^	2.86 ± 0.15^A^	tr	136.65 ± 6.20^A^	0.82 ± 0.01^B^	4.90 ± 0.21^C^	63.23 ± 2.45^E^	262.09 ± 10.09^B^	6.73 ± 0.31^A^	566.60 ± 25.03^B^	2.04 ± 0.08^B^	7.55 ± 0.22^A^
*C. horizontalis*	tr	0.69 ± 0.01^B^	2.28 ± 0.10^E^	4.80 ± 0.25^D^	tr	174.10 ± 5.40^B^	1.37 ± 0.05^C^	8.68 ± 0.43^E^	38.38 ± 2.10^B,C^	294.05 ± 13.01^B,C^	13.25 ± 0.60^C^	1012.83 ± 45.02^D^	tr	26.05 ± 1.00^C^
*C. nanshan*	nd	0.65 ± 0.01^B^	1.73 ± 0.10^C,D^	4.33 ± 0.20^C,D^	0.65 ± 0.01^A^	168.68 ± 6.40^B^	2.16 ± 0.12^D^	8.22 ± 0.38^D,E^	87.58 ± 3.54^F^	384.72 ± 18.12^D^	17.30 ± 0.75^D^	736.79 ± 30.01^C^	3.89 ± 0.20^D^	19.25 ± 0.95^B^
*C. hjelmqvistii*	tr	1.12 ± 0.04^C^	1.96 ± 0.10^D^	4.48 ± 0.32^D^	nd	174.55 ± 8.00^B^	0.56 ± 0.03^A^	3.36 ± 0.16^A,B^	41.19 ± 2.05^C^	335.10 ± 14.10^C^	17.09 ± 0.71^D^	1216.27 ± 50.01^E^	0.56 ± 0.02^A^	24.38 ± 1.05^C^
*C. dielsianus*	6.11 ± 0.20^C^	1.77 ± 0.03^E^	1.58 ± 0.08^C^	4.14 ± 0.25^B,C,D^	0.59 ± 0.02^A^	177.81 ± 6.43^B^	3.15 ± 0.16^E^	4.14 ± 0.19^B,C^	37.85 ± 1.04^B,C^	273.21 ± 15.02^B^	16.76 ± 0.80^D^	643.22 ± 15.15^B,C^	0.79 ± 0.03^A^	19.91 ± 0.55^B^
*C. splendens*	tr	1.55 ± 0.04^D^	2.80 ± 0.15^F^	5.59 ± 0.32^E^	nd	212.60 ± 11.00^C^	2.18 ± 0.11^D^	8.39 ± 0.50^D,E^	32.95 ± 1.14^A,B^	473.70 ± 20.01^E^	18.34 ± 0.61^D^	1225.89 ± 30.12^E^	2.49 ± 0.11^C^	37.30 ± 1.85^E^
*C. bullatus*	tr	0.51 ± 0.01^A^	1.53 ± 0.04^C^	3.73 ± 0.22^B,C^	0.51 ± 0.03^A^	120.49 ± 5.20^A^	2.37 ± 0.10^D^	2.54 ± 0.12^A^	29.83 ± 1.10^A^	215.22 ± 10.00^A^	10.85 ± 0.55^B^	677.53 ± 16.15^C^	4.07 ± 0.15^D^	30.33 ± 1.10^D^
*C. zabelii*	3.25 ± 0.11^B^	1.44 ± 0.05^D^	1.08 ± 0.05^B^	3.61 ± 0.15^B^	nd	226.45 ± 5.40^C^	1.44 ± 0.06^C^	7.58 ± 0.35^D^	53.09 ± 2.70^D^	649.73 ± 25.05^F^	30.34 ± 1.32^E^	1690.23 ± 55.01^F^	5.78 ± 0.21^E^	9.75 ± 0.20^A^

^a^Values presented as means ± SD calculated per dw of the plant material (*n* = 3); tr—trace, the content less than 0.5 mg/100 g dw; nd—not detected; different capital letters within the same row indicate significant differences at *α* = 0.05 in HSD Tukey's test; 6 : 0—caproic acid, 8 : 0—caprylic acid, 12 : 0—lauric acid, 14 : 0—myristic acid, 15 : 0—pentadecylic acid, 16 : 0—palmitic acid, 17 : 0—margaric acid, 16 : 1 Δ^9^—palmitoleic acid, 18 : 0—stearic acid, 18 : 1 Δ^9^—oleic acid, 20 : 0—arachidic acid, 18 : 2 Δ^9,12^—linoleic acid, 20 : 1 Δ^11^—eicosenoic acid and 22 : 0—behenic acid.

**Table 2:** Content of phytosterols and triterpenes (mg/100 g dw) in the *Cotoneaster* fruits.^a^

Fruit sample	Campesterol	*β*-Sitosterol	Stigmasterol	*β*-Amyrin	*α*-Amyrin	Ursolic acid	Oleanolic acid
*C. lucidus*	6.83 ± 0.30^C^	195.31 ± 5.31^B^	nd	nd	1.05 ± 0.05^A^	6.61 ± 0.30^B^	15.52 ± 0.53^D^
*C. divaricatus*	9.06 ± 0.31^E^	132.19 ± 4.23^A^	nd	nd	2.48 ± 0.07^B^	2.21 ± 0.04^A^	8.65 ± 0.32^A,B^
*C. horizontalis*	6.04 ± 0.22^B,C^	316.31 ± 15.03^D^	nd	nd	0.88 ± 0.02^A^	25.45 ± 1.10^F^	17.24 ± 0.50^E^
*C. nanshan*	8.94 ± 0.40^E^	391.26 ± 17.02^E^	nd	nd	5.26 ± 0.21^C^	6.04 ± 0.22^B^	26.52 ± 1.05^F^
*C. hjelmqvistii*	4.31 ± 0.12^A^	211.99 ± 10.13^B^	nd	1.17 ± 0.05^A^	14.37 ± 0.61^F^	27.03 ± 0.98^F^	18.41 ± 0.50^E^
*C. dielsianus*	5.38 ± 0.21^B^	181.96 ± 5.22^B^	tr	2.12 ± 0.10^B^	6.32 ± 0.24^D^	10.49 ± 0.35^C^	7.30 ± 0.18^A^
*C. splendens*	13.11 ± 0.56^F^	463.26 ± 15.10^F^	nd	nd	8.79 ± 0.30^E^	13.42 ± 0.45^D^	17.05 ± 0.45^D,E^
*C. bullatus*	7.98 ± 0.31^D^	274.47 ± 12.15^C^	2.70 ± 0.07^B^	0.88 ± 0.04^A^	14.15 ± 0.50^F^	41.45 ± 1.50^G^	13.05 ± 0.52^C^
*C. zabelii*	6.77 ± 0.30^C^	273.25 ± 10.22^C^	1.00 ± 0.01^A^	nd	14.89 ± 0.22^F^	20.70 ± 1.03^E^	9.27 ± 0.36^B^

^a^Values presented as means ± SD calculated per dw of the plant material (*n* = 3); tr—trace, the content less than 0.5 mg/100 g dw; nd—not detected; different capital letters within the same row indicate significant differences at *α* = 0.05 in HSD Tukey's test.

**Table 3:** UHPLC-PDA-ESI-MS^3^ data of polyphenols identified in the polar extracts from *Cotoneaster* fruits.

Number	Compounds	*t*_*R*_	UV	(M-H)^−^*m*/*z*	MS/MS *m*/*z* (% base peak)	CL	CDV	CHR	CN	CH	CDL	CS	CB	CZ
						%^b^								
1	Vanillic acid-hexoside	3.5	250, 290	329	MS^2^: 167 (100); 123 (2); 107 (4)	3.8	3.1	3.4	3.4	2.5	1.4	2.3	0.9	1.7
2	Unidentified	4.4	250, 295	255	MS^2^: 165 (23)	33.9	3.1	2.1	16.1	5.7	5.0	2.6	3.1	3.2
3	3-*O*-Caffeoylquinic acid	6.0	294, 325	353	MS^2^: 191 (100); 179 (47); 135 (6)	1.8	10.4	5.3	15.1	5.1	5.1	8.1	5.7	1.9
4	3-*O*-*p*-Coumaroylquinic acid	9.4	285, 310	337	MS^2^: 163 (100); 119 (10)	2.1	5.0	4.0	2.3	1.9	2.4	3.7	3.3	2.5
5	Caffeic acid hexoside	9.8	290, 323	341	MS^2^: 179 (100); 135 (10)	2.3	8.2	2.7	5.1	1.2	0.6	2.5	1.0	2.8
6	Unidentified	10.0	285, 323	439	MS^2^: 391 (100); 338 (17); 243 (10); 195 (55)	3.9	1.9	4.3	4.8	2.3	1.2	2.0	1.3	1.3
7	5-*O*-Caffeoylquinic acid (chlorogenic acid)^a^	10.4	294, 325	353	MS^2^: 191 (100); 179 (6)	29.4	28.3	29.5	26.0	23.5	23.0	17.9	17.3	10.8
8	4-*O*-Caffeoylquinic acid	10.9	294, 325	353	MS^2^: 191 (21); 179 (47); 173 (100)	1.1	4.1	2.4	5.1	1.0	1.4	2.4	2.1	2.0
9	Procyanidin B-type dimer	13.7	280	577	MS^2^: 451 (30); 425 (100); 407 (55); 289 (10) MS^3^ (425): 407 (80); 273 (13)	0.7	1.6	0.5	1.2	1.1	1.2	0.9	nd	0.7
10	Procyanidin B-2^a^	14.9	280	577	MS^2^: 451 (25); 425 (100); 407 (62); 289 (14); MS^3^ (425): 407 (95); 273 (9)	0.3	2.5	4.6	0.8	8.0	6.2	5.5	9.3	10.2
11	5-*O*-*p*-Coumaroylquinic acid	15.7	285, 310	337	MS^2^: 191 (100); 163 (7)	11.7	3.1	1.2	3.6	1.2	0.6	0.8	1.4	1.9
12	(−)-Epicatechin^a^	16.4	280	289	MS^2^: 245 (100); 205 (28)	2.4	4.4	8.5	2.1	15.8	12.3	9.7	18.6	34.4
13	Procyanidin B-type dimer	17.3	280	577	MS^2^: 451 (25); 425 (100); 407 (45); 289 (6); MS^3^ (425): 407 (75); 273 (9)	nd	nd	nd	nd	0.4	nd	0.6	0.9	1.2
14	Procyanidin B-type tetramer	18.3	280	1153	MS^2^: 1027 (15); 863 (80); 739 (15); 501 (05); 491 (58); 289 (100)	nd	nd	nd	nd	1.0	nd	0.6	1.1	1.4
15	Procyanidin C-1^a^	20.6	280	865	MS^2^: 847 (19); 739 (77); 713 (51); 695 (100); 577 (26); MS^3^ (713): 695 (100); 561 (30); 543 (31); 425 (32); 407 (36)	0.5	1.9	3.2	nd	5.3	3.7	3.3	5.6	7.3
16	Procyanidin B-type tetramer	23.3	280	1153	MS^2^: 863 (90); 739 (10); 501 (65); 491 (62); 289 (100)	nd	nd	2.1	nd	2.7	2.2	2.0	2.7	3.5
17	Quercetin 3-*O*-*β*-D-(2^″^-*O*-*β*-D-xylosyl)galactoside^a^	23.9	268, 355	595	MS^2^: 463 (10); 445 (14); 300 (85); MS^3^ (463): 343 (62); 301 (100)	nd	nd	4.0	nd	2.0	2.6	16.1	5.9	nd
18	Epicatechin derivative	26.2	280	739	MS^2^: 587 (100); 451 (19); 339 (40); 289 (35)	nd	2.2	2.0	nd	1.1	nd	1.5	1.2	nd
19	Unidentified	26.3	280	451	MS^2^: 341 (100); 217 (8)	nd	2.5	2.6	2.4	1.5	2.0	2.0	1.2	1.2
20	Quercetin rhamnoside-hexoside	26.7	275, 350	609	MS^2^: 301 (100)	0.6	0.4	1.4	nd	0.7	3.2	2.3	0.9	1.8
21	Quercetin 3-*O*-*β*-D-galactoside (hyperoside)^a^	27.1	265, 355	463	MS^2^: 301 (100)	2.5	5.0	4.9	5.5	5.5	9.5	5.2	6.6	2.4
22	Querectin 3-*O*-*β*-D-(6^″^-*O*-α-L-Rhamnosyl)glucoside (rutin)^a^	27.3	260, 355	609	MS^2^: 301 (100)	0.8	2.5	2.6	2.8	3.8	2.5	2.0	nd	2.2
23	Quercetin 3-*O*-*β*-D-glucoside (isoquercitrin)^a^	28.0	265, 355	463	MS^2^: 301 (100)	1.6	3.1	2.4	2.4	3.5	2.5	3.3	2.6	3.2
24	Procyanidin B-type dimer	28.6	280	577	MS^2^: 425 (100); 407 (52); 289 (18)	0.6	1.6	2.0	1.0	1.6	2.0	1.6	2.0	2.4
25	Quercetin rhamnoside-hexoside	31.3	276, 350	609	MS^2^: 301 (100)	nd	2.2	2.2	nd	0.4	4.1	nd	2.9	nd
26	Quercetin 3-*O*-*α*-L-rhamnoside (quercitrin)^a^	32.4	276, 350	447	MS^2^: 301 (100)	nd	2.8	2.2	nd	1.4	5.1	1.8	2.6	nd

^a^Identified with the corresponding standards; ^b^relative contribution based on peak area on the UHPLC chromatograms (*λ* = 280 nm) recorded at the extract concentration of 10 mg/mL and injection volume of 3 *μ*L; nd—not detected; the values are means (*n* = 3); with RSD ≤ 5%. CL, *C. lucidus*; CDV, *C. divaricatus*; CHR, *C. horizontalis*; CN, *C. nanshan*; CH, *C. hjelmqvistii*; CDL, *C. dielsianus*; CS, *C. splendens*; CB, *C. bullatus*; CZ, *C. zabelii*.

**Table 4:** Total phenolic content (TPC) and antioxidant activity (DPPH, FRAP, and TBARS tests) of the *Cotoneaster* fruits and standard antioxidants.

Fruit sample/standard	TPC^a^ (mg GAE/g)	Radical scavenging activity DPPH^b^		Reducing power^c^		LA-peroxidation TBARS^d^	
		EC_50_ (*μ*g/mL)	TE (*μ*mol TE/g)	FRAP (mmol Fe^2+^/g)	TE (*μ*mol TE/g)	IC_50_ (*μ*g/mL)	TE (*μ*mol TE/g)
*C. lucidus*	28.70 ± 1.01^B^	123.41 ± 1.70^E^	122.75 ± 1.69^C^	0.70 ± 0.01^B^	257.22 ± 4.96^B,C^	108.70 ± 4.11^F^	314.84 ± 6.03^C^
*C. divaricatus*	29.71 ± 0.91^B^	91.47 ± 2.01^C^	165.58 ± 3.62^D^	0.76 ± 0.01^C^	281.61 ± 4.43^C^	83.16 ± 0.58^D^	406.94 ± 1.43^D^
*C. horizontalis*	30.50 ± 0.72^B^	93.32 ± 1.90^C^	162.38 ± 3.31^D^	0.85 ± 0.01^D^	322.75 ± 4.06^D^	84.89 ± 2.11^D^	401.23 ± 5.03^D^
*C. nanshan*	26.02 ± 0.74^A^	178.35 ± 2.81^F^	84.91 ± 1.33^B^	0.61 ± 0.01^A^	213.41 ± 4.42^A^	165.76 ± 3.74^G^	205.30 ± 2.33^B^
*C. hjelmqvistii*	43.50 ± 1.21^D^	64.51 ± 0.84^B^	234.84 ± 2.91^E,F^	1.05 ± 0.02^F^	414.38 ± 11.14^F,G^	62.96 ± 1.10^C^	532.92 ± 4.63^E,F^
*C. dielsianus*	31.02 ± 1.02^B^	117.10 ± 2.40^D^	129.37 ± 2.65^C^	0.67 ± 0.03^B^	240.90 ± 13.83^A,B^	103.72 ± 2.58^E^	322.66 ± 3.98^C^
*C. splendens*	38.51 ± 0.81^C^	67.15 ± 1.80^B^	225.49 ± 6.04^E^	0.98 ± 0.01^E^	383.06 ± 6.24^E,F^	66.21 ± 2.94^C^	518.18 ± 11.79^E^
*C. bullatus*	37.31 ± 0.80^C^	66.31 ± 1.70^B^	228.54 ± 5.86^E^	0.97 ± 0.01^E^	378.87 ± 2.90^E^	64.99 ± 1.55^C^	523.90 ± 6.30^E,F^
*C. zabelii*	43.02 ± 1.11^D^	62.93 ± 1.91^B^	240.93 ± 7.28^F^	1.09 ± 0.04^G^	434.27 ± 20.50^G^	62.54 ± 1.32^C^	543.86 ± 5.76^F^
QU	—	1.70 ± 0.11^A^	8.96 ± 0.58^A^	31.20 ± 0.98^K^	11878.15 ± 15.20^J^	1.85 ± 0.12^A^	18.37 ± 1.69^A^
BHA	—	2.90 ± 0.15^A^	5.24 ± 0.27^A^	16.14 ± 0.77^I^	7726.31 ± 10.52^H^	3.16 ± 0.22^A^	10.76 ± 1.06^A^
BHT	—	6.50 ± 0.13^A^	2.34 ± 0.05^A^	18.89 ± 0.45^J^	9247.66 ± 12.30^I^	9.31 ± 0.16^B^	3.64 ± 0.09^A^
TX	—	3.80 ± 0.20^A^	—	9.34 ± 0.35^H^	—	8.47 ± 0.45^B^	—

^a–d^Results expressed as means ± SD calculated per dw of the plant material (*n* = 3); different capital letters within the same row indicate significant differences at *α* = 0.05 in HSD Tukey's test. ^a^Total phenolic content (TPC), expressed in gallic acid equivalents (GAE). ^b^Scavenging efficiency in the DPPH test, the amount of the plant materials or standards required for 50% reduction of the initial DPPH concentration expressed as EC_50_, effective concentration. ^c^Ferric reducing antioxidant power. ^d^Ability to inhibit linoleic acid (LA) peroxidation monitored by TBARS test and expressed as IC_50_, concentration of plant materials or standards needed to decrease the LA-peroxidation by 50%; TE, Trolox® equivalent antioxidant activity. Standards: QU, quercetin; BHA, butylated hydroxyanisole; BHT, 2,6-di-*tert*-butyl-4-methylphenol; TX, Trolox®.

**Table 5:** Inhibitory effects of *Cotoneaster* fruit extracts and standards towards lipoxygenase (LOX) and hyaluronidase (HYAL).

Fruit sample/standard	LOX		HYAL	
	IC_50_^a^ (*μ*g/mL)	IC_50_^b^ (*μ*g/U)	IC_50_^a^ (*μ*g/mL)	IC_50_^b^ (*μ*g/U)
*C. lucidus*	487.75 ± 6.57^F^	13.29 ± 0.18^F^	25.65 ± 0.95^C^	16.44 ± 0.61^C^
*C. divaricatus*	479.98 ± 12.79^F^	13.08 ± 0.35^F^	34.22 ± 1.48^D^	21.93 ± 0.95^D^
*C. horizontalis*	421.85 ± 5.78^E^	11.50 ± 0.16^E^	40.51 ± 2.11^E,F,G^	25.97 ± 1.35^E,F,G^
*C. nanshan*	626.16 ± 5.04^H^	17.07 ± 0.14^H^	45.64 ± 0.76^G^	29.25 ± 0.49^G^
*C. hjelmqvistii*	290 ± 2.75^C^	7.70 ± 0.07^C^	44.44 ± 1.72^F,G^	28.48 ± 1.10^F,G^
*C. dielsianus*	914.97 ± 2.15^J^	24.94 ± 0.06^J^	35.07 ± 2.60^D,E^	22.48 ± 1.66^D,E^
*C. splendens*	734.25 ± 5.86^I^	20.01 ± 0.16^I^	34.36 ± 0.11^D^	22.03 ± 0.07^D^
*C. bullatus*	585.43 ± 16.14^G^	15.96 ± 0.44^G^	39.04 ± 0.82^D,E,F^	25.03 ± 0.53^D,E,F^
*C. zabelii*	375.87 ± 9.89^D^	9.97 ± 0.26^D^	33.33 ± 2.12^D^	21.37 ± 1.36^D^
QU	69.60 ± 2.62^A^	2.46 ± 0.01^A^	21.04 ± 1.03^C^	13.87 ± 0.06^C^
ECA	124.38 ± 1.56^B^	3.39 ± 0.04^B^	18.51 ± 0.50^B^	11.87 ± 0.32^B^
CHA	151.71 ± 7.52^B^	4.14 ± 0.21^B^	20.35 ± 0.36^B^	13.05 ± 0.23^B^
IND	90.12 ± 0.40^A^	1.89 ± 0.10^A^	8.61 ± 0.22^A^	5.60 ± 0.07^A^

Results expressed as means ± SD calculated per dry weight (dw) of the extracts; different capital letters within the same row indicate significant differences at *α* = 0.05 in HSD Tukey's test. Standards: QU, quercetin; ECA, (−)-epicatechin; CHA, chlorogenic acid; IND, indomethacin. Ability to inhibit lipoxygenase (LOX) and hyaluronidase (HYAL) calculated as the amount of analyte needed for 50% inhibition of enzyme activity was expressed as follows: ^a^*μ*g of the dry extracts or standards/mL of the enzyme solution and ^b^*μ*g of the extracts/enzyme units (U).

## Data Availability

The data used to support the findings of this study are available from the corresponding author upon request.
